# Deciphering the mechanism of *Peptostreptococcus anaerobius*-induced chemoresistance in colorectal cancer: the important roles of MDSC recruitment and EMT activation

**DOI:** 10.3389/fimmu.2023.1230681

**Published:** 2023-09-13

**Authors:** Jinhua Gu, Xiaojun Lv, Wenwen Li, Guangcai Li, Xialian He, Ye Zhang, Lihong Shi, Xiaoqian Zhang

**Affiliations:** ^1^ Department of Gastroenterology, Affiliated Hospital of Weifang Medical University, Weifang, China; ^2^ School of Clinical Medicine, Weifang Medical University, Weifang, China; ^3^ College of Rehabilitation Medicine, Weifang Medical University, Weifang, China

**Keywords:** *Peptostreptococcus anaerobius*, chemoresistance, MDSCs, IL-23, EMT, colorectal cancer

## Abstract

*Peptostreptococcus anaerobius* (*P. anaerobius*, *PA*) in intestinal flora of patients with colorectal cancer (CRC) are associated with poor prognosis. Studies have shown that *P. anaerobius* could promote colorectal carcinogenesis and progression, but whether *P. anaerobius* could induce chemoresistance of colorectal cancer has not been clarified. Here, both *in vitro* and *in vivo* experiments showed that *P. anaerobius* specifically colonized the CRC lesion and enhanced chemoresistance of colorectal cancer to oxaliplatin by recruiting myeloid-derived suppressor cells (MDSCs) into the tumor microenvironment. Furthermore, this study revealed that it was the increased secretion of IL-23 by MDSCs that subsequently facilitated the epithelial–mesenchymal transition (EMT) of tumor cells to induce chemoresistance of CRC by activating the Stat3-EMT pathway. Our results highlight that targeting *P. anaerobius* might be a novel therapeutic strategy to overcome chemoresistance in the treatment of CRC.

## 1 Introduction

Colorectal cancer (CRC) is the third most common diagnosed cancer and the second-leading cause of cancer death worldwide. Moreover, in the past few decades, CRC is shifting to diagnosis at a younger age and a more advanced stage (1–3). Despite the fact that palliative chemotherapy for advanced-stage colorectal cancer has led to substantial improvement of overall survival, over half of CRC patients suffered from chemoresistance, and the pervasive development of acquired chemoresistance has always been the main cause of cancer relapse and metastasis (4–6). Therefore, uncovering the underlying mechanisms associated with CRC chemoresistance is indispensable for designing novel treatment strategies.

The intestinal flora, representing the largest microbial reservoir in human body, is intimately associated with human growth, nutritional metabolism, and disease onset (7–9). The cecum and colon harbor the most dense and diverse communities of bacteria in gut microhabitats. These bacteria can be found in feces, gut lumen, colon mucus layers, colorectal epithelia, and even tumor stroma (10, 11). Meanwhile, it is remarkable that the intestinal flora has been found to be involved in regulating the onset and progression of CRC by modulating the tumor microenvironment (12). It is reported that some intestinal flora, such as *Streptococcus bovis*, *Enterotoxigenic Bacteroides fragilis*, and *Enterococcus faecalis*, can promote the occurrence, development, and chemoresistance of CRC through inflammatory reaction, genotoxins, oxidative stress, metabolites, and biofilms (11). In particular, certain bacteria such as *Gamma-proteobacteria* and *F. nucleatum* can penetrate mucus and lead to chemoresistance by metabolizing chemotherapeutics and activating autophagy in colorectal tumor (13, 14). *Peptostreptococcus anaerobius* (*P. anaerobius*, *PA*), an anaerobic Gram-positive bacterium that commonly exists in human oral and intestinal tracts, has been found in high abundance in intestinal flora of chemoresistant CRC patients (13, 15–18) and *P. anaerobius* could directly educate CRC cells and the corresponding microenvironment to promote cancer progression (13, 17–19). However, whether *P. anaerobius* could induce CRC chemoresistance and, if so, its underlying mechanism, remains unclear.

Recruited from immature myeloid cells by tumor-derived growth factors and inflammatory factors (20–22), MDSCs play important roles in modulating immune responses to promote CRC progression (20). CRC patients with high levels of MDSCs have worse outcomes (23–27) than those with low levels of MDSCs (28–30). Remarkably, a significantly high enrichment of MDSCs in a CRC model with *P. anaerobius-*treated ApcMin/+mice was reported recently (19). Consistently, another prognostic analysis showed that *P. anaerobius* was enriched in high-risk stage III colon cancer samples, and the invaded bacteria activated tumor-associated myeloid cells and caused them to produce the cytokine IL-23, which was significantly characteristic in the high-risk group (31). These findings indicated that both *P. anaerobius* and MDSCs were closely related to the development of CRC, but the relationships among *P. anaerobius*, MDSCs, and chemoresistance still need to be further clarified.

**Figure 1:**
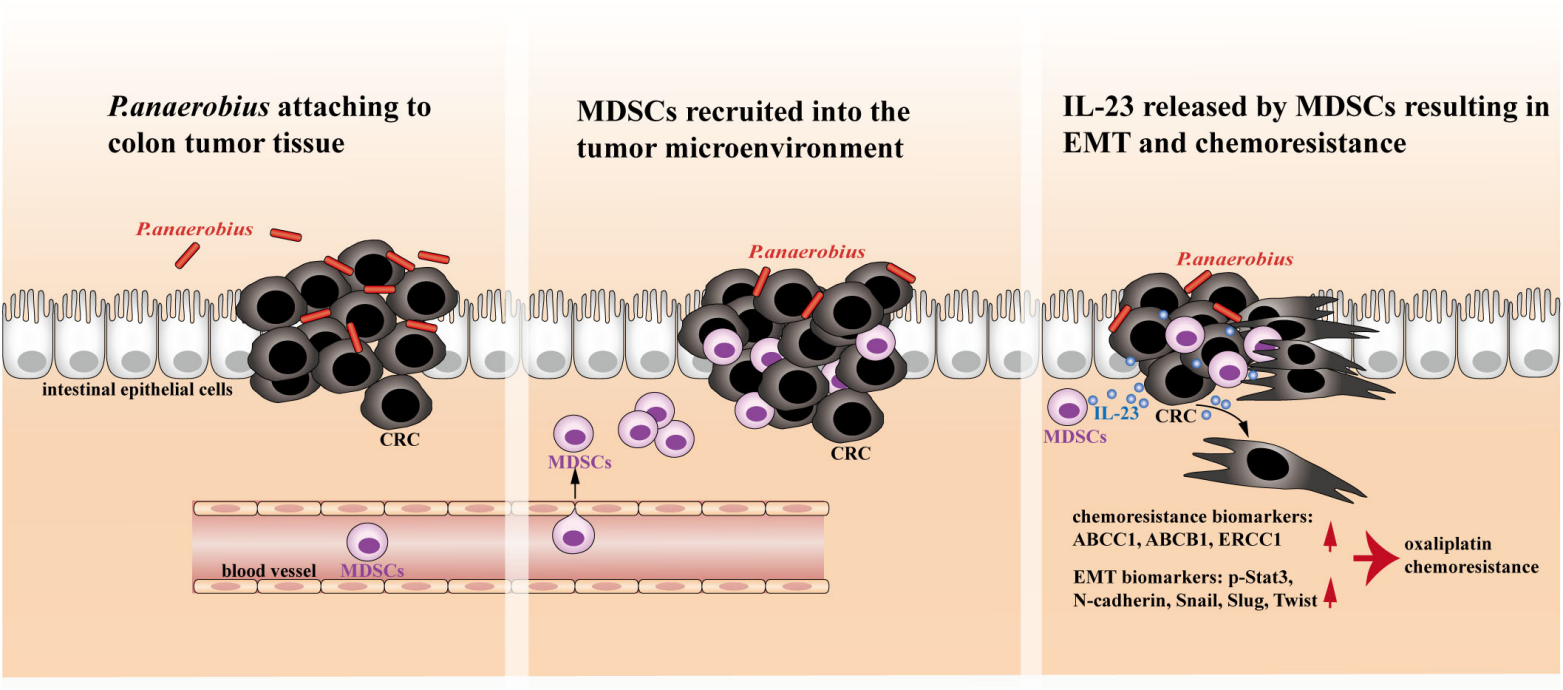
The proposed mechanistic scheme of *P. anaerobius* promoting colorectal chemoresistance.

In this study, both *in vitro* and *in vivo* experiments demonstrated that *P. anaerobius* could promote chemoresistance of CRC to oxaliplatin by colonizing colorectal tumor lesion and facilitating the recruitment of MDSCs into the tumor microenvironment, which drove EMT and chemoresistance of tumor cells by releasing IL-23 (**Figure 1**).

## 2 Materials and methods

### 2.1 Bacterial culture

*P. anaerobius* (ATCC27337) was purchased from Ningbo Mingzhou Biotechnology Co. Ltd (B81243, MingZhouBio). The bacteria were maintained in Modified Reinforced Clostridial Broth Medium (MD039; ATCC Medium 2107, Shandong Topu Biol-Engineering Co. Ltd) in an anaerobic jar (D-110, MITSUBISHI). The anaerobic condition was created by the usage of Anaeropack (D-04, AN0035; MGC AnaeroPackTM Series, MITSUBISHI).

### 2.2 Animal experiments

**Figure 2:**
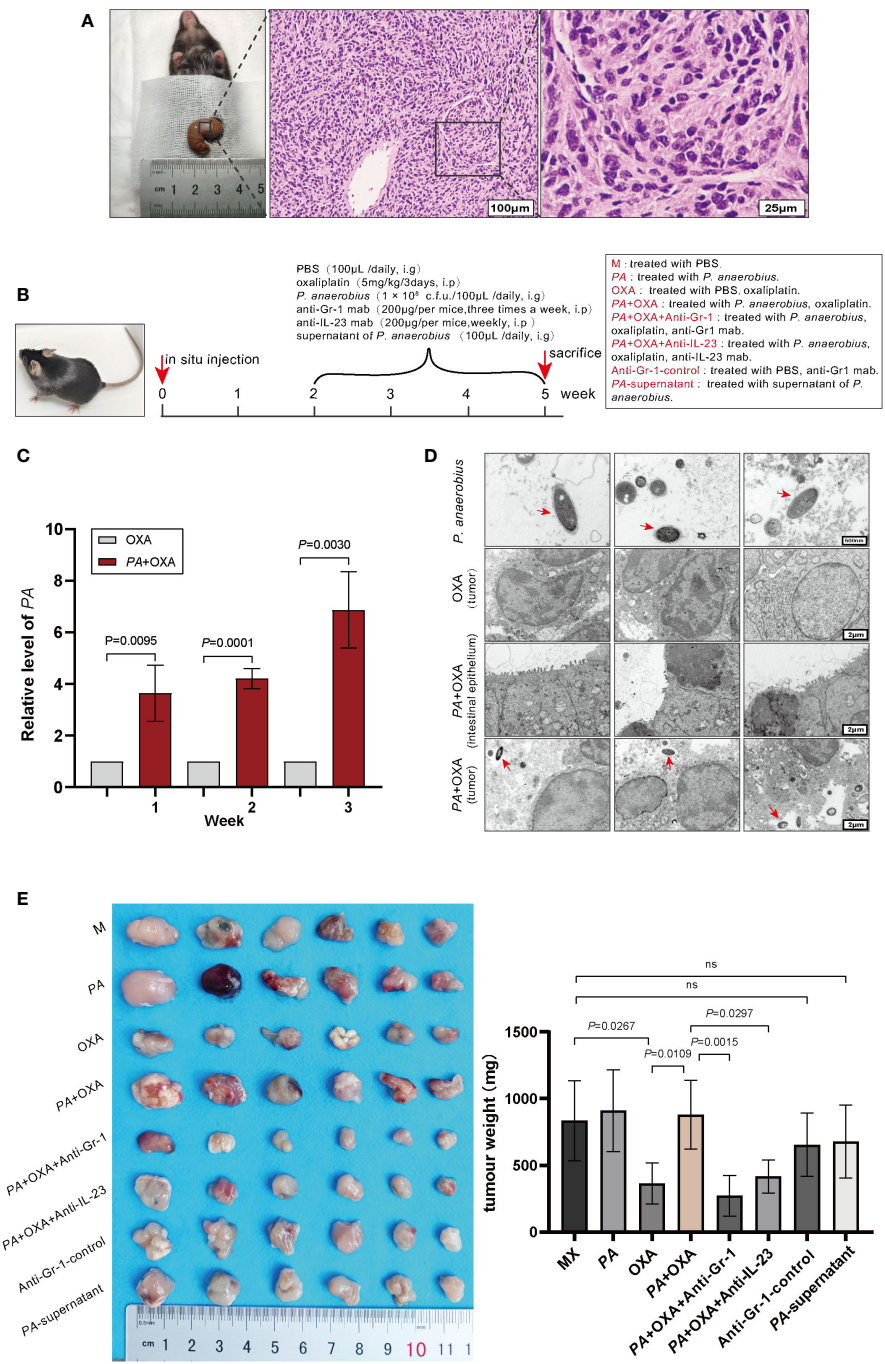
*P. anaerobius* attenuated the therapeutic effect of oxaliplatin in CRC mice. **(A)** Representative morphologies and histological images (H&E) of CRC tissues. Scale bar: 100 µm (left), 25µm (right). **(B)** Schematic diagram of experimental design and timeline of CRC mice model (*n* = 6). *PA*: *P. anaerobius*; OXA: oxaliplatin; anti-Gr-1mab: anti-mouse Gr-1 monoclonal antibody; anti-IL-23 mAb: anti-mouse IL-23 monoclonal antibody. **(C)** The amount of *P. anaerobius* in stool samples of CRC mice determined by qPCR (mean ± SD, two-tailed unpaired Student’s *t*-test). **(D)** Representative TEM images of *P. anaerobius* (red arrows) attaching to colon tumor tissue. Scale bars: 500 nm for *PA* images, 2 μm for colon tumor tissue images. **(E)** Representative tumor images and statistical analysis of tumor weights of CRC in different groups (*n* = 6, mean ± SD, one-way analysis of variance).

MC-38 cells (1 × 10^6^ cells per mouse) were implanted in the cecum of C57BL/6 mice (male, 8 weeks old). Two weeks after implantation, mice were gavaged with *P. anaerobius* suspension (1 × 10^8^ c.f.u.) concurrently with administration of oxaliplatin (5 mg/kg/3 days) intraperitoneally for 3 weeks, and feces were collected weekly for qPCR analysis (**Figure 2B**). In addition, anti-Gr-1 monoclonal antibody (32) (anti-Gr-1 mAb, 200 μg/mouse, three times/week, BE0075, Bio X cell) or anti-mouse IL-23 monoclonal antibody (anti- IL-23 mAb, 200 μg/mouse/week, BE0313, Bio X cell) was intraperitoneally given to the corresponding group of mice respectively for 3 weeks to observe the function of MDSCs and IL-23 in *P. anaerobius-*induced chemoresistance. Then, the mice were sacrificed and colonic tumors were collected and weighed. All animal work was approved by the Animal Experimentation Ethics Committee of Weifang Medical University.

### 2.3 Assessment of colonic histopathology

Colonic tumor specimens were formalin-fixed and paraffin-embedded for histologic examination. Sections of 5 μm were stained with H&E and reviewed in a blinded manner by an experienced pathologist. Dysplasia was defined according to the latest World Health Organization Classification of Tumors of the Digestive System.

### 2.4 Microbial DNA extraction and *P. anaerobius* quantification

Stool DNA was extracted by ZR Fecal DNA MiniPrep (D2700, Beijing Solarbio Science & Technology Co., Ltd) from the feces samples of mice. DNA was quantified using a Nanodrop 2000 spectrophotometer (Thermo Fisher Scientific, Waltham, MA). Two microliters of DNA (0.5 ng) was used in each 20 μL of 2×SYBR Green qPCR Master Mix (G3320, Servicebio) reaction. The reaction was performed in triplicate and analyzed on a QuantStudio 7 Flex System (CFX Connect, Bio-Rad). The primers for *P. anaerobius* were GTA AAG GGT GCG TAG GTG GTC (forward 5’→3’) and CCT CAG TGT CAG TTG CAG TCC (reverse 5’→3’), and primers for total bacteria were GTG STG CAY GGY TGT CGT CA (forward5’→3’) and ACG TCR TCC MCA CCT TCC TC (reverse 5’→3’).

### 2.5 Transmission electron microscopy

Tumor tissues were fixed in 1% OsO_4_ in 0.1 MPB (pH 7.4) and rinsed three times in 0.1 MPB (pH 7.4). After that, the samples were dehydrated, embedded, cut into 50-nm sections, and stained with 2% uranium acetate and 2.6% lead citrate. A transmission electron microscope (HT7800/HT7700, HITACHI) was used to obtain corresponding images.

### 2.6 Flow cytometry

Multicolor flow cytometry (FCM) was performed to observe the percentage of MDSCs in the bone marrow of CRC mice. After being freed of muscles and tendons, the femurs and tibiae of mice were placed in 70% ethanol for 2 min and subsequently washed in PBS, then a syringe was used to flush bone marrow cells from the femurs and tibias with PBS. After red blood cells were lysed, flushing fluid was filtered through a 100-μm membrane to obtain suspension of single cells. Cells were incubated with Fc blocking antibody (BioLegend, 101319) for 15 min and then stained with fluorescence-conjugated antibodies of surface markers CD11b (clone M1/70, eBioscience, 11-0112-82) and Gr-1 (clone RB6-8C5, Biogems, 83122-80-25) for 30 min. The samples were detected by a BD FACS Aria Fusion Flow Cytometry Cell Sorter (BD Biosciences), and the data were analyzed using FlowJo v.9 software (FlowJo LLC).

### 2.7 Immunofluorescence

Slides (3–4 µm thick) of colonic tumor specimens were prepared and incubated with FITC-conjugated anti-mouse CD11b (BioLegend, USA, 101205) and APC-conjugated anti-mouse Gr1 (BioLegend, USA, 101211) overnight at 4°C. Images were acquired using a fluorescence microscope (Olympus, Japan). Quantification of fluorescent signals was performed using ImageJ software. The density of infiltrated MDSC in the tumor microenvironment was evaluated by averaged CD11b^+^Gr1^+^ co-positive (red and green) area from at least three random 0.42 mm^2^ fields within the tumors.

### 2.8 Cell culture

The colon cancer cell line MC-38 was obtained from ATCC and cultured in the usual culture medium composed of RPMI-1640 medium (GIBCO, Carlsbad, CA) and 10% fetal bovine serum (FBS) at 37°C in a humidified 5% CO_2_ atmosphere. For bacterial co-culture, MC-38 cells were exposed to *P. anaerobius* with a multiplicity of infection (MOI) of 200 for 6 h under anaerobic conditions. Then, the medium containing *P. anaerobius* was replaced with the usual medium supplemented with 2% penicillin/streptomycin and 10 mg/mL gentamicin. After 24 h, conditioned medium was collected and named *PA*+MC-38-CM for further research.

### 2.9 Wright’s Giemsa staining

Naive MDSCs collected through flow cytometry were prepared by cytospin to perform morphological assessment using Wright–Giemsa (Leagene, Beijing, China) staining according to the manufacturer’s protocol.

### 2.10 Migration and invasion assay

Transwell assays for evaluating migration and the invasion ability of cells were conducted using 24-well Millicell Hanging Cell Culture Insert 8.0 µm PET (Merck Millipore, Darmstadt, Germany). For migration assay of MDSCs, 2 × 10^5^ cells per well were incubated in serum-free 1640 in the upper chamber with usual culture medium in lower wells supplemented with *PA*-CM (*PA* supernatant), MC-38-CM (MC-38 supernatant), or *PA*+MC-38-CM, respectively. For invasion assay of MC-38 cells (1 × 10^5^ cells per well), 8.0-µm PETs were coated with 10% Matrigel matrix to imitate extracellular matrix and the usual culture medium in lower wells was supplemented with MDSCs-CM (supernatant of MDSCs cultured with *PA*-MC-38-CM), IL-23 (40 ng/mL, ab259423, Abcam), or anti-IL-23 (100 ng/mL, BE0313, Bio X cell) + MDSCs-CM, respectively. After 48 h, migrating/invasion cells on the basolateral side of the chamber membrane were fixed with formaldehyde and stained with crystal violet (Merck Millipore, Darmstadt, Germany). The number of migrating/invading cells was counted under a light microscope at a magnification of ×400 in five random fields. All assays were repeated at least three times independently.

### 2.11 For wound-healing assay

MC-38 cells were pretreated with MDSCs-CM, IL-23 (40 ng/mL, ab259423, Abcam) or anti-IL-23 (100 ng/mL, BE0313, Bio X cell) + MDSCs-CM for 24 h respectively. The wound-healing assay was performed by scratching a single cell layer with a pipette tip. Images of the scratch area were recorded at five random spots at 0 and 48 h. The migration distance of the wound edge was measured using a standard size field for each image. The mean migration distances of the five spots were calculated in triplicate and all data were statistically analyzed.

### 2.12 ELISA

Proteins were extracted from the tumor tissues as described previously (33). The expression levels of VEGF, HGF, IL-6, and IL-23 in MDSCs culture supernatant with corresponding stimulations and the IL-23 level in tumor tissues were analyzed by commercial ELISA kits (R&D Systems) according to the manufacturer’s protocol. The color reaction was measured as OD_450_ units on the microplate reader (Model 550; Bio-Rad). The concentration of cytokines was determined via a standard curve that was obtained using the kit’s standards. Experiments were performed in triplicate.

### 2.13 Cell viability assay

The viability of MC-38 cells was evaluated by the CCK-8 (BS350B, Biosharp) assay. Co-culture of *P. anaerobius* and MC-38 cells or *PA*-CM treatment were conducted to observe the influence of *P. anaerobius* on the efficacy of OXA (0.1360 μM) to MC-38 cells (5 × 10^3^ cells per well). In addition, the efficacy of OXA (0.1360 μM) to MC-38 cells treated with MDSCs-CM, IL-23, and anti-IL-23+MDSCs-CM (100 ng/mL, BE0313, Bio X cell) respectively, were also detected. After cells (5 × 10^3^ cells per well) were incubated for 24 h, CCK-8 assay was conducted by adding 10 μL of CCK-8 reagent to each well and incubating for 3 h. Finally, the optical density was determined at 450 nm using the microplate reader (Model 550; Bio-Rad). Experiments were performed at least in triplicate.

### 2.14 Western blot

The proteins were isolated from cells, separated by 10% SDS–PAGE, and transferred onto PVDF membranes. Then, the membranes were blocked with 5% BSA for 2 h and incubated with primary antibodies overnight at 4°C and secondary antibodies for 1 h at room temperature, respectively. Anti-ABCC1 (1:1,000, bs-24241R), Anti-ABCB1 (1:1,000, bs-0563R), Anti-ERCC1 (1:1,000, bs-1726R), Snail (1:2,000, bs-1371R), and Twist (1:2,000, bs-2441R) were obtained from Bioss (Bioss, BeiJing); N-cadherin (1:1,000, 14215S), E-cadherin (1:1,000, 14472S), Slug (1:2,000, 9585T), Stat3 (1:1,000, 9139T), and p-Stat3 (1:1,000, 4113S) were obtained from Cell Signaling Technology (MA); β-actin (1:5,000, 81115-1-RR) was obtained from Proteintech (MA); Goat-anti-mouse IgG (1:5,000, A0216) was obtained from beyotime (Shanghai). Membranes were exposed to Pierce ECL Western Blotting Substrate (GE Healthcare). Band intensities were determined using ImageJ (National Institutes of Health). The band intensities were represented by the averages of three independent experiments.

### 2.15 Statistical analysis

A Student’s *t*-test was performed to compare the variables of the two sample groups. Multiple group comparisons were made by one-way analysis of variance (ANOVA) followed by Tukey’s test. *p*-value less than 0.05 was considered statistically significant. Data were expressed as mean ± SD from three independent experiments. All tests were performed using GraphPad Prism, version 8.0 (GraphPad, La Jolla, CA) or SPSS, version 20 (SPSS Inc, Chicago, IL).

## 3 Results

### 3.1 *Peptostreptococcus anaerobius* accumulated in implanted colon cancer lesion and attenuated the therapeutic effect of oxaliplatin

To investigate the roles of *P. anaerobius* in CRC chemoresistance, a colorectal cancer model in C57 mice was constructed by implanting MC-38 cells *in situ*. Two weeks later, three randomly selected mice were dissected to observe tumor growth and all reached 80–100 mm^3^ tumor volume (**Figure 2A**). Then, *P. anaerobius* (1 × 10^8^ c.f.u.) were gavaged to CRC mice daily accompanied with oxaliplatin treatment (5 mg/kg/3 days) for 3 weeks (**Figure 2B**). Quantitative PCR proved that *P. anaerobius* were successfully colonized in intestinal flora (**Figure 2C**) and transmission electron microscopy (TEM) showed that *P. anaerobius* was more likely to accumulate in the colon cancer lesion than in normal intestinal epithelium (**Figure 2D**). In addition, it seemed that the accumulation of *P. anaerobius* could promote the growth of implanted colon cancer since the weight of CRC treated with *P. anaerobius* was higher than that without *P. anaerobius* treatment. Interestingly, the supernatant of *P. anaerobius* had no obvious influence on tumor proliferation (**Figure 2E**). Furthermore, *P. anaerobius* significantly hindered the effectiveness of oxaliplatin while the growth of implanted colon cancer without *P. anaerobius* gavage could be effectively inhibited by oxaliplatin (**Figure 2E**), indicating that *P. anaerobius* could attenuate the therapeutic effect of oxaliplatin in CRC mice.

### 3.2 *Peptostreptococcus anaerobius* promoted drug resistance by recruiting MDSCs into colorectal cancer microenvironment

**Figure 3:**
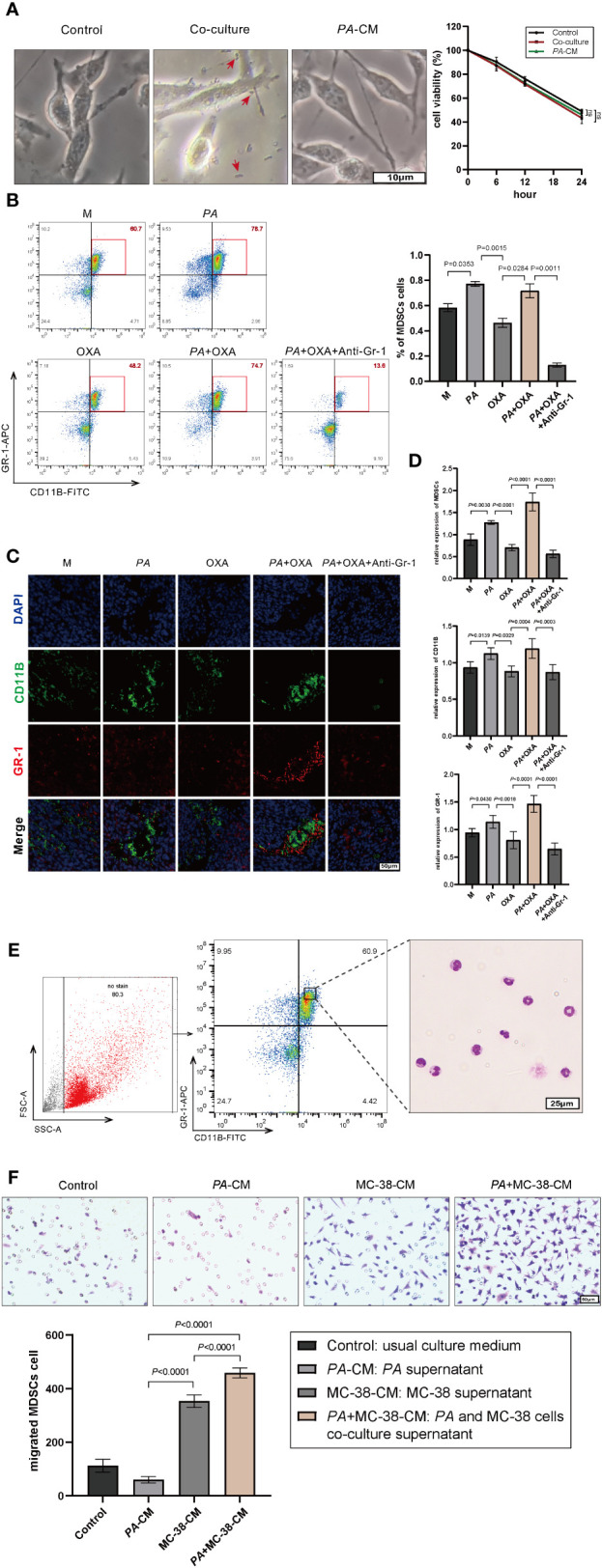
*P. anaerobius* promoted oxaliplatin resistance by recruiting MDSCs into the CRC microenvironment. **(A)** Viability of MC-38 cells co-cultured with *P. anaerobius* or in conditioned medium supplemented with *PA* supernatant (CCK-8 assay, *p >* 0.05, *PA* indicated by red arrow). **(B)** MDSCs (Gr-1^+^CD11B^+^) from bone marrow of CRC mice detected by multicolor flow cytometry. **(C, D)** MDSCs infiltrated into the tumor microenvironment detected by fluorescein isothiocyanate (FITC) with anti-mouse CD11b antibody (green), allophycocyanin (APC), anti-mouse Gr-1 antibody (red), and DAPI (blue). **(E)** Morphological feature of MDSCs collected by FCM (Giemsa staining, purple-blue leaf-shaped or round-type nucleus and almost colorless cytoplasm). **(F)** Chemotaxis ability of MDSCs treated with *PA*-CM, MC-38-CM, and *PA*+MC-38-CM (co-culture medium of *PA* and MC-38), respectively. **(A–F)** Data were presented as mean ± SD, *p-*values were determined by one-way analysis of variance. Three independent experiments were performed with consistent results.

As having been reported that the accumulation of *P. anaerobius* in colorectal cancer lesion was closely related to chemoresistance (19, 31), this study also found that *P. anaerobius* could attenuate the therapeutic effect of oxaliplatin in a mouse model. Meanwhile, *in vitro* cell experiments showed that either the co-culture of MC-38 and *PA* or *PA* supernatant stimulation did not affect the sensitivity of MC-38 to oxaliplatin (**Figure 3A**). Since MDSCs have been reported to be modulated by *P. anaerobius* and be responsible for developing chemoresistance (21), the MDSCs in bone marrow of CRC mice were analyzed by flow cytometry. The result showed that the amount of MDSCs was significantly higher in bone marrow of CRC mice treated with *P. anaerobius* (**Figure 3B**). In addition, immunofluorescence showed a significant increase of MDSC infiltration in colorectal tumor lesions of mice treated with *P. anaerobius* (**Figures 3C, D**). These findings are consistent with previously reported studies that found increasing proportion of MDSCs in CRC with *P. anaerobius* infection (19). Furthermore, MDSCs collected from the bone marrow of CRC mice (**Figure 3E**) were incubated by *PA*-CM, MC-38-CM, or *PA*+MC-38-CM, respectively. Interestingly, the chemotaxis ability of MDSCs treated with *PA*+MC-38-CM increased significantly while compared with those treated with MC-38-CM and *PA*-CM (**Figure 3F**), indicating that it was the interaction between *P. anaerobius* and MC-38 but not the metabolites of *P. anaerobius* that induced the infiltration of MDSCs into the tumor microenvironment.

Anti-Gr-1 mAb can selectively cut down MDSCs (34) and has no obvious influence on other immune cells, then anti-Gr-1 mAb (200 μg/mouse, three times/week) was intraperitoneally injected in mice to eliminate MDSCs both in the bone marrow (**Figure 3B**) and in the tumor microenvironment (**Figures 3C, D**). It was interesting to find out that the tumor was evidently diminished while MDSCs were eliminated by Anti-Gr-1 in the *PA*+OXA+Anti-Gr-1 group compared with the *PA*+OXA group (**Figure 2E**), and tumor was also diminished in the Anti-Gr-1-control group compared with the M group; however, there was no statistically significant difference. This could be attributed to the lower levels of MDSCs in the M group, and the fact that Anti-Gr-1 mAb does not directly exert cytotoxic effects on the tumor. Taken together, these data suggested that *P. anaerobius* could facilitate chemoresistance by promoting the recruitment of MDSCs into the colorectal cancer microenvironment.

### 3.3 IL-23 secreted by MDSCs promoted chemoresistance of CRC cells

**Figure 4:**
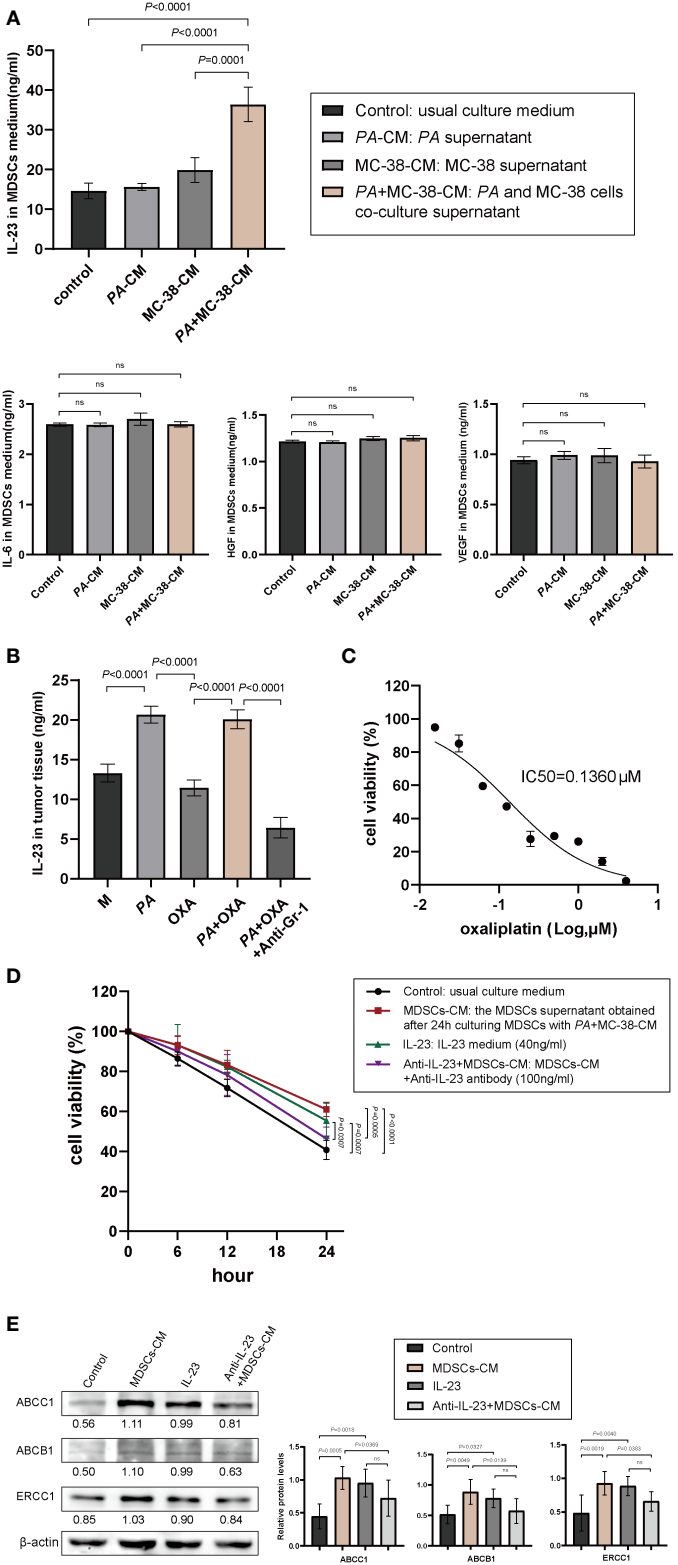
IL-23 released by MDSCs promoted chemoresistance of CRC. **(A)** The expression levels of IL-6, VEGF, HGF, and IL-23 in culture medium of MDSCs determined by ELISA. **(B)** IL-23 levels in tumor tissues with different treatments. **(C)** The viability of MC-38 cells treated with different concentrations of oxaliplatin (CCK-8 assay). **(D)** The viability of MC-38 cells treated with oxaliplatin after pretreatment with MDSCs-CM, recombinant IL-23, or anti-IL-23+MDSCs-CM, respectively. **(E)** The expression of chemoresistance biomarkers of ABCB1, ABCC1, and ERCC1 determined by Western blotting (mean ± SD, one-way analysis of variance, triplicated).

It has been reported that elevated MDSCs could contribute to tumor progression by remodeling TME through autocrine and paracrine (35–37), and a number of soluble factors secreted by MDSCs, such as IL-6, IL-23, HGF, and VEGF, in various tumors including CRC were associated with poor chemotherapeutic effect (31). Therefore, IL-6, IL-23, HGF, and VEGF in the culture medium of MDSCs were detected by ELISA, and the results showed that the IL-23 level of MDSCs treated with *PA*+MC-38-CM increased significantly and was the highest in all groups (**Figure 4A**). In addition, a similar result was obtained *in vivo* that IL-23 in tumor tissues of CRC mice treated with *P. anaerobius* was significantly higher than that of the CRC model mice (**Figure 4B**). Furthermore, IL-23 in tumor tissues decreased remarkably after MDSCs were eliminated by anti-Gr-1 mAb (**Figure 4B**).

Next, the role of IL-23 on CRC chemoresistance was investigated. CCK-8 assay was used to test the viability of MC-38 cells treated with oxaliplatin, and the IC_50_ of oxaliplatin to MC-38 cells was 0.136 μM (**Figure 4C**). As expected, both MDSCs-CM and recombinant IL-23 boosted chemoresistance of MC-38 cells to oxaliplatin, and interestingly, anti-IL-23 antibody attenuated MDSCs-CM induced chemoresistance of MC-38 cells to oxaliplatin (**Figure 4D**). Consistent with the results of CCK-8 assay, Western blot analysis also showed obviously increased expression of chemoresistance biomarkers (ABCB1, ABCC1, and ERCC1) in MC-38 cells stimulated by MDSCs-CM or IL-23 while anti-IL-23 antibody dramatically diminished the expression of ABCB1, ABCC1, and ERCC1 in MDSCs-CM-treated MC-38 cells (**Figure 4E**). Moreover, the tumor weight was significantly reduced by intravenous administration of anti-IL-23 antibody (**Figure 2E**). These results strongly suggested that IL-23 secreted by MDSCs promoted chemoresistance of CRC cells to oxaliplatin.

### 3.4 IL-23 promotes chemoresistance by activating the EMT in colorectal cancer cells

**Figure 5:**
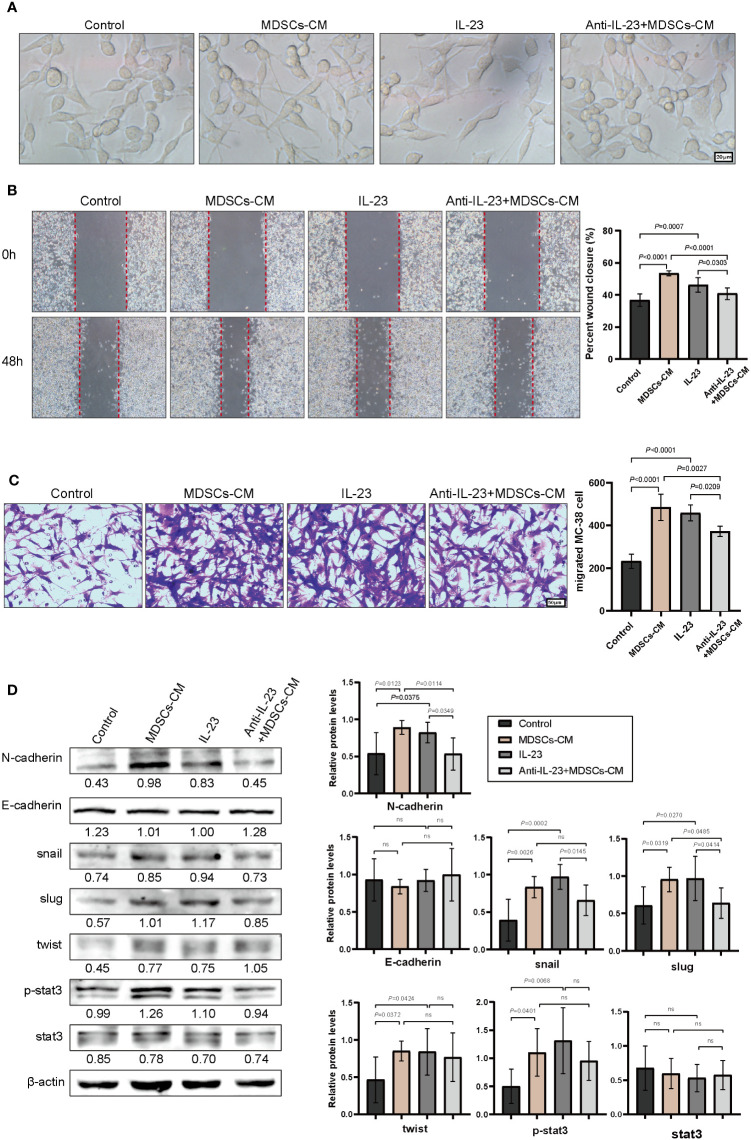
IL-23 activated the EMT signaling pathway. **(A)** The morphology of MC-38 cells after incubation (48 h) with MDSCs-CM, IL-23, anti-IL-23+MDSCs-CM, respectively. **(B)** Representative images of wound healing assay of MC-38 cells. **(C)** Representative images of invasive MC-38 cells. **(D)** The expression of N-cadherin, E-cadherin, Snail, Slug, Twist, Stat3, and P-stat3 in MC-38 cells detected by Western blot. **(B–D)** Data were presented as the mean ± SD; *p-*values were determined by one-way analysis of variance. Three independent experiments were performed with consistent results.

Subsequently, the underlying mechanism of chemoresistance induced by interaction of *P. anaerobius* and colorectal cancer was investigated. Epithelial–mesenchymal transition (EMT) has always been a major cause of chemoresistance in various kinds of cancers (38) and notable mesenchymal-like fusiform morphological changes were observed in MC-38 cells treated with MDSCs-CM (**Figure 5A**); thus, wound-healing assay and transwell invasion assay were carried out to observe the migration and invasion ability of MC-38 cells. The results showed that MDSCs-CM and IL-23 could significantly enhance the migration and invasion ability of MC-38 cells, and this enhancement could be inhibited by anti-IL-23 antibody (**Figures 5B, C**). Furthermore, Western blot analysis showed that N-cadherin, Snail, Slug, Twist, and p-Stat3 expression were significantly upregulated in MDSCs-CM-treated MC-38 cells, whereas the expression of E-cadherin had no obvious change (**Figure 5D**). In conclusion, these findings suggested that IL-23, which is secreted by MDSCs, could promote chemoresistance in colorectal cancer cells by activating EMT.

## 4 Discussion

Metagenomic profiling of stool and mucosal samples from CRC patients revealed that *P. anaerobius* was an oncogenic bacterial candidate enriched in CRC (13, 17, 18) and *P. anaerobius* has been found to be involved in the proliferation and chemoresistance of CRC (19). However, the underlying mechanisms about the contribution of *P. anaerobius* to CRC chemoresistance remains unknown. In this study, we elucidated that the accumulation of *P. anaerobius* in tumor lesion could mediate the recruitment of MDSCs into the CRC microenvironment and promote IL-23 secretion by MDSCs, which led to EMT and chemoresistance of CRC cells.

Bacterial colonization, such as *F. nucleatum* and *γ-proteobacteria*, are often prerequisite steps to tumor malignant progression (14, 39). Consistent with previous reports, *P. anaerobius* avidly colonized the implanted colon tumor lesions in C57 mice and attenuated the effectiveness of oxaliplatin. Considering that *P. anaerobius* has not been found to induce CRC chemoresistance directly and the modification of the tumor immune microenvironment has been reported to play vital roles in intestinal bacteria-related drug resistance, we suspected that *P. anaerobius* might promote chemoresistance by modulating the CRC microenvironment.

Since MDSCs have been reported to be modulated by *P. anaerobius* and responsible for developing chemoresistance (40, 41), the MDSCs in bone marrow and in colorectal tumor lesions of CRC mice were analyzed. The results showed that the amount of MDSCs both in bone marrow and in implanted colon tumor lesions was significantly increased in CRC mice infected with *P. anaerobius*. Furthermore, *in vitro* experiments showed that *PA*+MC-38-CM had the highest ability of enhancing chemotaxis ability of MDSCs among *PA*-CM and MC-38-CM. In addition, the sensitivity of implanted colorectal cancer to oxaliplatin was rescued by MDSC elimination. All these findings indicated that *P. anaerobius* might facilitate chemoresistance by the aggregation of MDSCs into the colorectal cancer microenvironment and the interaction between *P. anaerobius* and colorectal cancer cells contributed to chemoresistance of CRC.

MDSCs promote tumor progression and chemoresistance by remodeling the tumor microenvironment via crosstalk with surrounding cells by expression of pro-inflammatory cytokines, growth factors, and angiogenic factors favoring tumor progression (21). Here, the main tumor-promoting cytokines released by MDSCs, VEGF, HGF, IL-6, and IL-23 were detected, and the results showed that only IL-23 levels were significantly increased in the supernatant of MDSCs cultured with *PA*+MC-38-CM as well as in implanted colorectal cancer loaded with *P. anaerobius*. Meanwhile, it is also important to note that IL-23 in implanted tumor tissues decreased remarkably and the efficacy of oxaliplatin significantly improved after MDSCs were eliminated by anti-Gr-1 mAb, suggesting that IL-23 released by MDSCs facilitated chemoresistance of CRC to oxaliplatin.

As EMT plays important roles in promoting stem cell transformation and chemoresistance (38) and IL-23R was abundantly expressed in colorectal cancer cells (42), we wondered if IL-23 mediated the chemoresistance and EMT of colorectal cancer. As expected, both MDSCs-CM and IL-23 induced increased expression of chemoresistance and mesenchymal biomarkers as well as transcription factors Snail, Slug, and Twist by activating the Stat3-EMT signaling pathway, while this activation could be diminished by anti-IL-23 antibody, supporting the notion that both MDSC recruitment and IL-23 secretion are essential for *P. anaerobius-*related chemoresistance.

There is a complex interaction between tumor microbiome and gut microbiome, which leads to the limited effect of chemotherapy and a negative impact on the host immune system (43). Although *P. anaerobius* had negative roles in colorectal cancer progression, it could augment anti-tumor immune responses in oral squamous cell carcinoma (44). Anyway, the limitations of this study should be addressed. Firstly, the precise mechanisms by which *P. anaerobius* recruited MDSCs into CRC microenvironment were not fully elucidated in the current study. Secondly, a recent study found that MDSCs-derived IL-1β was involved in CRC chemoresistance (45), indicating the heterogeneity and necessity of epigenetic profiling for individualized diagnosis and treatment of cancer. Hence, we cannot dismiss the possibility that there might be additional cytokines contributing to the augmentation of chemoresistance in colorectal cancer. Further investigation is warranted to elucidate the specific mechanism through which MDSCs promote chemoresistance.

In conclusion, this study identified the potential contribution of *P. anaerobius* to colorectal cancer chemoresistance. In particular, the colonization of *P. anaerobius* in CRC lesion mediated the recruitment of MDSCs into the colorectal cancer microenvironment, which secreted IL-23 and subsequently promoted chemoresistance by activating Stat3-EMT of colon cancer cells. These findings provide clinical implications for improving prognostic assessment and designing new targeted treatment for CRC patients.

## Data availability statement

The raw data supporting the conclusions of this article will be made available by the authors, without undue reservation.

## Ethics statement

The animal study was reviewed and approved by the Laboratory Animal Ethics Committee of Affiliated Hospital of Weifang Medical University. The study was conducted in accordance with the local legislation and institutional requirements.

## Author contributions

JG and XL performed statistical analysis and drafted the manuscript. WL, GL, and YZ critically revised and finalized the manuscript. JG and XH performed data analysis and interpretation. LS and XZ reviewed and edited the manuscript. JG designed the study and performed all experiments. All authors contributed to the article and approved the submitted version.
